# Reversible cardiac hypertrophy induced by PEG-coated gold nanoparticles in mice

**DOI:** 10.1038/srep20203

**Published:** 2016-02-01

**Authors:** Chengzhi Yang, Aiju Tian, Zijian Li

**Affiliations:** 1 Institute of Vascular Medicine, Peking University Third Hospital, Key Laboratory of Cardiovascular Molecular Biology and Regulatory Peptides, Ministry of Health, Key Laboratory of Molecular Cardiovascular Sciences, Ministry of Education and Beijing Key Laboratory of Cardiovasicular Receptors Research Beijing 100191, China

## Abstract

Gold nanoparticles (GNPs) are attracting more and more attention for their great potential value in biomedical application. Currently, no study has been reported on the chronic cardiac toxicity of GNPs after repeated administration. Here we carried out a comprehensive evaluation of the chronic cardiac toxicity of GNPs to the heart. Polyethylene glycol (PEG) -coated GNPs at three different sizes (10, 30 and 50 nm) or PBS was administrated to mice *via* tail vein for 14 consecutive days. Then the mice were euthanized at 2 weeks, 4 weeks or 12 weeks after the first injection. The accumulation of GNPs in the mouse heart and their effects on cardiac function, structure, fibrosis and inflammation were analysized. GNPs with smaller size showed higher accumulation and faster elimination. None of the three sizes of GNPs affected cardiac systolic function. The LVIDd (left ventricular end-diastolicinner-dimension), LVMass (left ventricular mass) and HW/BW (heart weight/body weight) were significantly increased in the mice receiving 10 nm PEG-GNPs for 2 weeks, but not for 4 weeks or 12 weeks. These results indicated that the accumulation of small size GNPs can induce reversible cardiac hypertrophy. Our results provide the basis for the further biomedical applications of GNPs in cardiac diseases.

The accelerating development of nanotechnology leads to a dramatic expansion of potential biomedical applications of nanomaterials. Nanoparticles have been widely employed in the treatment and diagnosis of many diseases^1^,^2^,^3^. In particular, gold nanoparticles (GNPs) hold great promise for biomedical application^4^,^5^. Due to their ease of synthesis and modification^6^, GNPs are emerging as an appealing platform for drug delivery systems^7^,^8^,^9^. Furthermore, GNPs shows unique optical properties arising from surface plasmon oscillation of free electrons^10^,^11^, which make them a promising candidate as photo thermal agents in hyperthermia and biological imaging. Therefore, GNPs have attracted much attention in the field of nanomedicine for diagnostic and therapeutic purposes^12^,^13^. Considering that GNPs may be administered to humans, assessing their safety is gaining more and more attention. There have been several studies concerning the toxic effects of GNPs on the liver, lung, kidney, brain and reproductive system^14^,^15^,^16^,^17^. However, studies on the effects of GNPs on the heart are very limited. Mohamed Anwar K Abdelhalim found that exposure of 10 and 20 nm GNPs for 3 and 7 days resulted in myocardial injury in the rat, while 50 nm GNPs were safe to rats^18^. A. Vinodhini reported that 17 to 29 nm proanthocyanidin-synthesized GNPs protected the heart from isoproterenol-induced myocardial injury in the rats^19^. Our previous work revealed that 13 nm PEG-coated GNPs have no obviously adverse effects on the heart within one week^20^. However, there is currently no comprehensive study on the chronic cardiac toxicity of GNPs after repeated administration.

In the present study, we evaluated chronic effects of GNPs on cardiac systolic function, hypertrophy, fibrosis and inflammation. The size of GNPs is a crucial physical parameter that affects their biodistribution and bioactivity^16^,^21^,^22^. Therefore, we carried out our study using three different sizes of GNPs (10, 30 and 50 nm). Besides, the surface functionalization of GNPs is another key factor that governs the safety of GNPs^23^. PEGylation, a strategy that employs polyethylene glycol (PEG) as modifying polymer, can increase blood half-life of GNPs and reduce their immunogenicity and antigenicity^1^,^24^,^25^. And the polyethylene glycol (PEG) polymer used for modifying drugs is approved by FDA and has yielded conjugates of great therapeutic and market success^1^. In fact, several studies concerning GNPs employed PEG as modifying polymer^14^,^16^,^26^. Thus, all the GNPs employed in our study are PEG-coated GNPs. The working flow chart is shown as Fig. 1A.

This study was to address the biological accumulation and safety of chronic GNPs exposure in the heart. The results indicated that the cardiac accumulation pattern of GNPs depends on their sizes after repeated administration in mice and the accumulation of 10 nm PEG-GNPs can induce reversible cardiac hypertrophy on weeks 2. These novel findings will help for the applications of GNPs in the disease diagnosis, drug delivery, disease therapy and the toxicity researches of other Nanoparticles.

## Results

### Characterization of the GNPs

**Figure 1:**
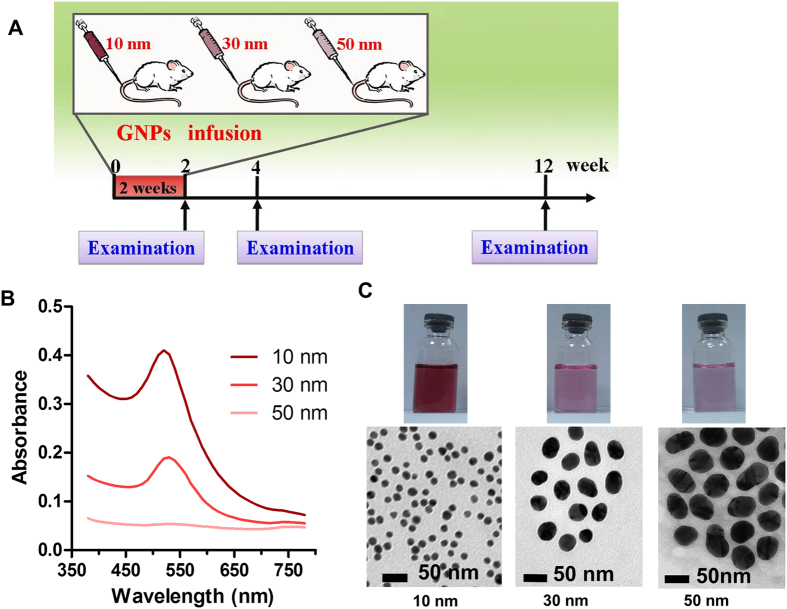
Treatment of BALB/c mice with GNPs and characterization of GNPs. (**A**) Scheme of the biodistribution and nanotoxicology experiment. (**B**) The wavelength absorption of three different sizes (10, 30 and 50 nm) of GNPs in the visible region of the optical spectrum. (**C**) Images of GNPs suspension and transmission electron microscopy (TEM) images of GNPs.

Previous studies have suggested that the biodistribution and toxicity effects of GNPs are size-dependent^16^,^22^. To investigate whether the size of GNPs affects their heart toxicity, three different sizes (10, 30 and 50 nm) of GNPs were employed in the present study. It’s well known that GNPs possess a strong surface plasmon resonance, which is manifested by an absorption band in the visible region of the optical spectrum^5^. As shown in Fig. 1B, the 10, 30 and 50 nm GNPs exhibited a maximum absorption wavelength around 520 nm, 530 nm and 535 nm respectively. These data were consistent with the bathochromic shift phenomenon of GNPs that absorption band changes to longer wavelength if the particle size is increased^27^. Owing to their different molar concentrations (10 nm GNPs > 30 nm GNPs > 50 nm GNPs), the 10 nm GNPs showed highest absorbance, followed by 30 nm GNPs and 50 nm GNPs. The absence of absorbance at wavelengths above 600 nm suggested that GNPs were well dispersed in the suspension. Furthermore, the morphology, size and aggregation state of the GNPs were evaluated using transmission electron microscopy (Fig. 1C).

### The accumulation of GNPs in the mouse heart

**Figure 2:**
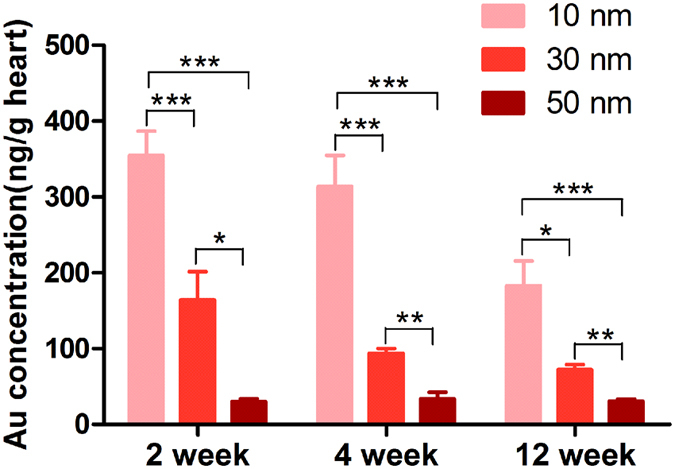
Au accumulation in heart tissues. The BALB/c mice received GNPs for 14 consecutive days. Then the Au content was determined with inductively coupled plasma mass spectrometry (ICP-MS) on weeks 2, 4 and 12 after the first administration. *P < 0.05, **P < 0.01, ***P < 0.001. Data represent means ± SEM.

The GNPs (400 μg/kg bodyweight) and vehicle control (PBS) were administered to mice intravenously *via* tail vein for 14 consecutive days. It was reported that GNPs accumulating in the heart were not completely eliminated up to 6 months after one dose injection^16^. To analyze the kinetics of GNPs in the heart, the Au concentration was determined by ICP-MS. The result showed that accumulation of GNPs in the heart was in a size-dependent manner at three time point (Fig. 2). The 10 nm GNPs always showed the highest accumulation at different time point. In addition, the accumulation of 10 nm and 30 nm GNPs decreased with time in the heart. The accumulation of 50 nm GNPs did not show overt change during the 12 weeks.

### Effects of the GNPs on cardiac function

**Figure 3:**
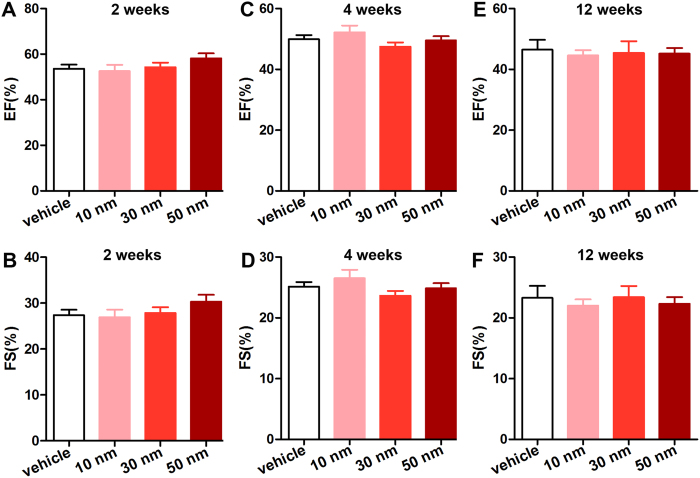
The effects of GNPs on cardiac function. Diastolic left ventricular posterior wall thickness (LVPW;d) and systolic left ventricular posterior wall thickness (LVPW;s) were measured. Ejection fraction (EF) and fractional shortening (FS) were calculated from these parameters. EF and FS were not significantly different among mice receiving vehicle, 10, 30 and 50 nm of PEG-coated GNPs on weeks 2 (**A**,**B**), 4 (**C**,**D**) and 12 (**E**,**F**).

The accumulation of GNPs in the heart raised the question whether they could adversely affect cardiac function. To assess the chronic effects of GNPs exposure on cardiac function, the two-dimensional echocardiography was performed. The left ventricular ejection fraction (EF) and fractional shortening (FS) have been most widely used to evaluate left ventricular systolic function. As shown in Fig. 3, the EF and FS of mice receiving 10, 30, or 50 nm GNPs were not significantly different from those of mice receiving vehicle at 2 weeks, 4 weeks and 12 weeks. These results indicated that the GNPs accumulated in the mouse heart did not affect cardiac systolic function.

### Effects of the GNPs on cardiac structure

**Figure 4:**
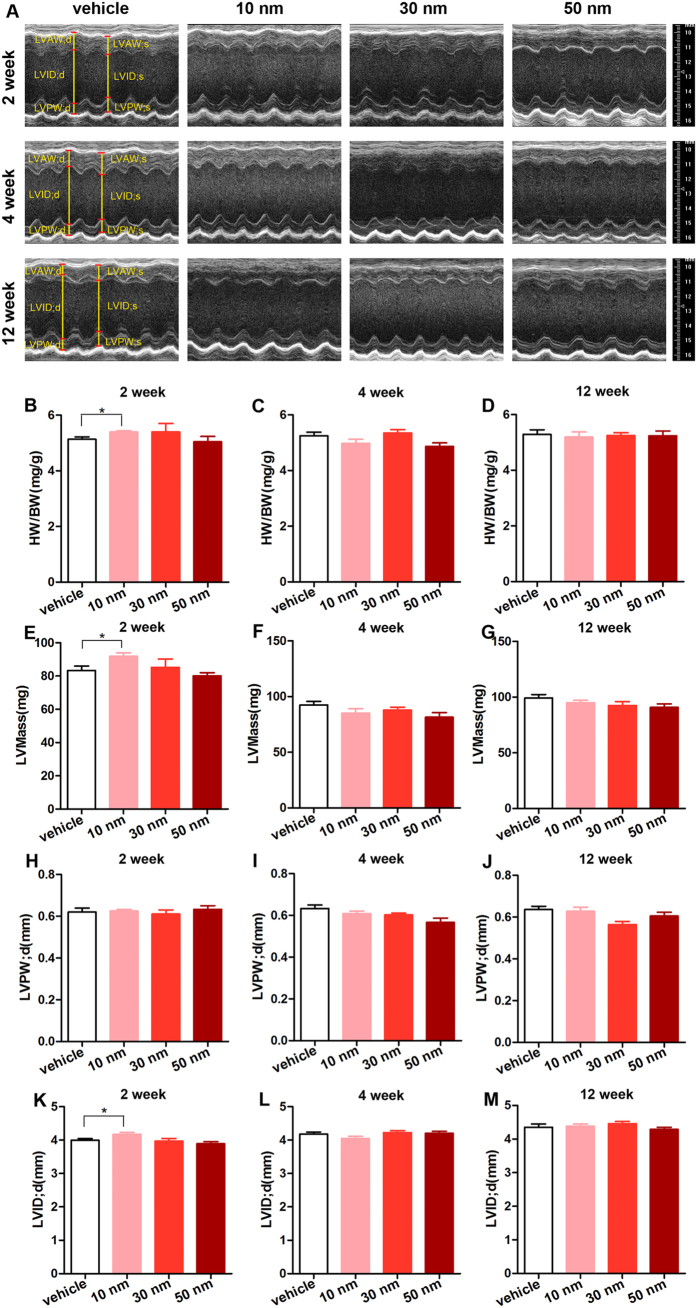
The effects of GNPs on cardiac size. The two-dimensional echocardiography was performed to analyse the cardiac structure *in vivo* at each time point (**A**). On weeks 2, 4, and 12, the body weight was measured before the mice were sacrificed. The heart weight was measured immediately the heart was excised. The heart weight to body weight (HW/BW) was calculated (**B**–**D**). The diastolic left ventricular posterior wall thickness (LVPW;d, (**H**–**J**)), systolic left ventricular posterior wall thickness (LVPW;s), diastolic left ventricular anterior wall thickness (LVPW;d), systolic left ventricular anterior wall thickness (LVPW;s), left ventricular end-diastolicinner-dimension (LVID;d, (**K**–**M**)) and left ventricular end- systolic inner-dimension (LVID;d) were measured. And the left ventricular mass (LVMass, (**E**–**G**)) were calculated from these parameters. *P < 0.05. Data represent means ± SEM.

Cardiac structure is essential to its function. Here we investigated the effects of GNPs on cardiac structure. The ratio of heart weight to body weight (heart weight/body weight, HW/BW) is the gold standard to evaluate cardiac structure. There was no significant difference in the HW/BW among the mice receiving 30 and 50 nm GNPs and the mice receiving vehicle at each time points (Fig. 4B–D). However, the mice receiving 10 nm GNPs for 2 weeks showed a statistically increase in HW/BW compared with mice receiving vehicle(5.39 ± 0.05 mg/g vs. 5.13 ± 0.08 mg/g, *P* < 0.05, Fig. 4B). To further analyze the cardiac structure in detail, the echocardiography was performed (Fig. 4A). The diastolic left ventricular posterior wall thickness (LVPWd), systolic left ventricular posterior wall thickness (LVPWs), left ventricular end-diastolic inner-dimension (LVIDd) and left ventricular end-systolic inner-dimension (LVIDs) were measured and left ventricular mass (LVMass) was calculated. Consistent with the HW/BW, there was a significant increase in LVMass in the mice that received 10 nm GNPs for 2 weeks compared with the vehicle group (91.89 ± 2.01 mg vs. 83.41 ± 2.64 mg, *P* < 0.05, Fig. 4E). The LVIDd was also found to increase significantly in the mice receiving 10 nm GNPs for 2 weeks compared with the vehicle group (4.17 ± 0.05 mm vs. 3.99 ± 0.05 mm, *P* < 0.05, Fig. 4K). However, the LVPWd among the four group mice was not significantly different (Fig. 4H–J). Taken together, these data indicate that injection of 10 nm GNPs for 2 weeks may result in reversible cardiac hypertrophy, while 30 and 50 nm GNPs did not affect cardiac size during 12 weeks.

### Effects of the GNPs on cardiac fibrosis

**Figure 5:**
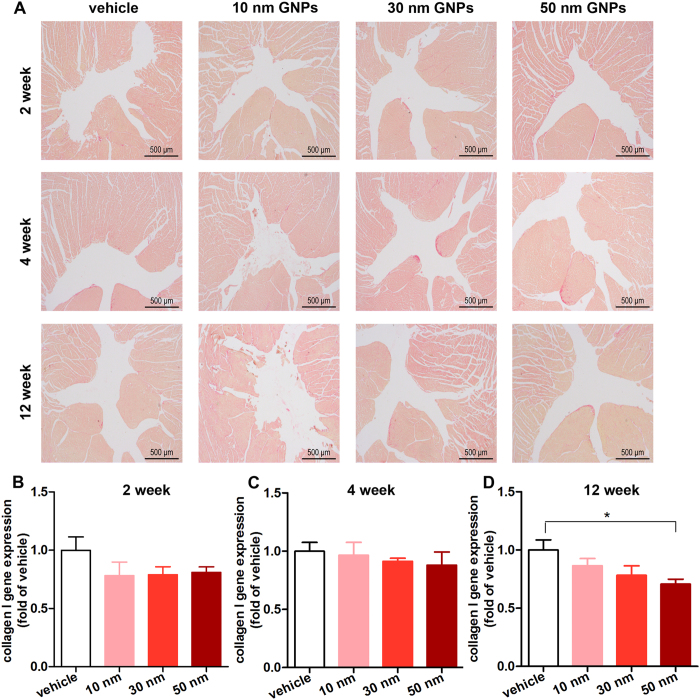
The effects of GNPs on cardiac fibrosis. (**A**) Representative micrographs of picrosirius red-stained sections of the ventricle. The yellow parts represent cardiac muscle and red parts represent collagen. Injection of 10, 30 and 50 nm GNPs did not result in cardiac fibrosis. All scale bars are 500 μm. The collagen I gene expression was detected with real-time PCR on weeks2 (**B**), weeks 4 (**C**) and weeks 12 (**D**). Fold changes were compared with vehicle group. *P < 0.05.

The extracellular fibrillar collagen plays a pivotal role in cardiac integrity and wound repair after injury. However, adverse accumulation of collagen results in cardiac fibrosis, which leads to ventricular diastolic and systolic dysfunction^28^. Therefore, the effect of the accumulated GNPs on cardiac collagen deposition is of great importance. The cardiac interstitial collagen was detected with Sirius red staining. As shown in Fig. 5A, no overt cardiac fibrosis was observed in mice receiving 10, 30 and 50 nm GNPs at each time point (Supplementary Fig. S1A shows the positive control of cardiac fibrosis). It has been accepted that type I collagen (collagen I) is the major constituent of the cardiac collagen^29^. To further investigate whether GNPs affect collagen synthesis, the collagen I gene expression was analyzed using real-time PCR. There was no significant change in the collagen I gene expression in mice receiving 10 and 30 nm GNPs. However, the mice receiving 50 nm GNPs exhibited a significant decrease in the collagen I gene expression at the 12th week (1.00 ± 0.09 vs. 0.71 ± 0.04, *P* < 0.05, Fig. 5D). These data suggested that exposure to 50 nm GNPs for 12 weeks may decrease the synthesis of collagen, while 10 and 30 nm GNPs did not affect the synthesis of collagen.

### Effects of the gold nanoparticles on cardiac inflammation

**Figure 6:**
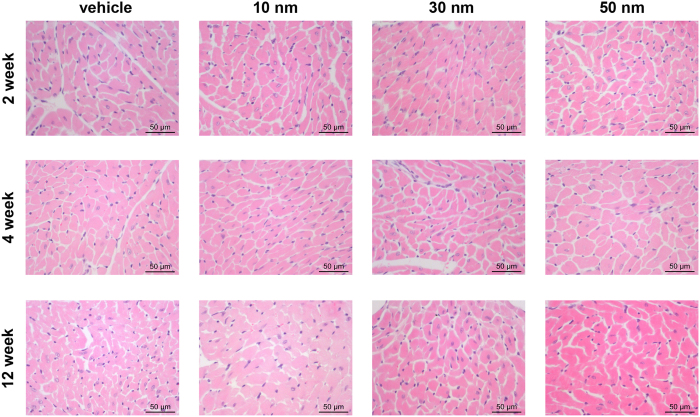
Representative images showing hematoxylin and eosin (HE) staining of heart sections from mice receiving vihicle, 10, 30 and 50 nm GNPs. All scale bars are 50 μm.

The cardiac inflammatory reaction is essential for cardiac repair, but it is also involved in pathologic cardiac remodeling^30^. Our previous study showed that 13 nm PEG-coated GNPs did not lead to acute cardiac inflammation^20^. However, it has not been unraveled whether chronic GNPs exposure of GNPs will result in cardiac inflammation. In this study we examined the effects of the gold nanoparticles on cardiac inflammation. It’s well accepted that inflammatory cell infiltration is the main feature of inflammation. First of all, hematoxylin and eosin (HE) staining was performed for morphological analysis of cardiac cells. Our data showed there was no obvious infiltration of blood cells in the heart sections of mice receiving GNPs (Fig. 6). Besides, no overt morphological alternations of cardiocytes were observed.

**Figure 7:**
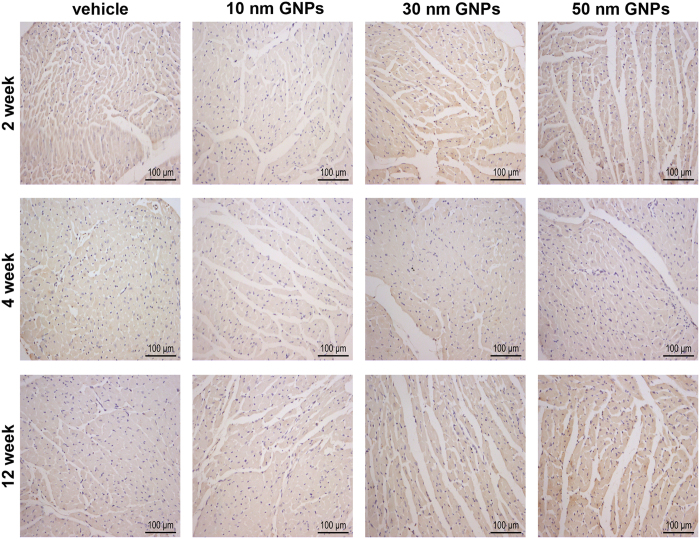
Representative micrographs showing Mac-3^+^ in the heart of mice receiving vihicle, 10, 30 and 50 nm GNPs. All scale bars are 100 μm.

**Figure 8:**
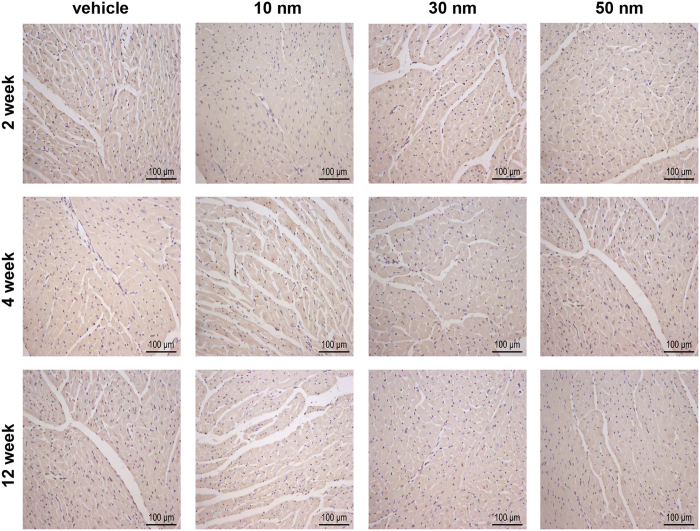
Representative micrographs showing CD45^+^ in the heart of mice receiving vihicle, 10, 30 and 50 nm GNPs. All scale bars are 100 μm.

**Figure 9:**
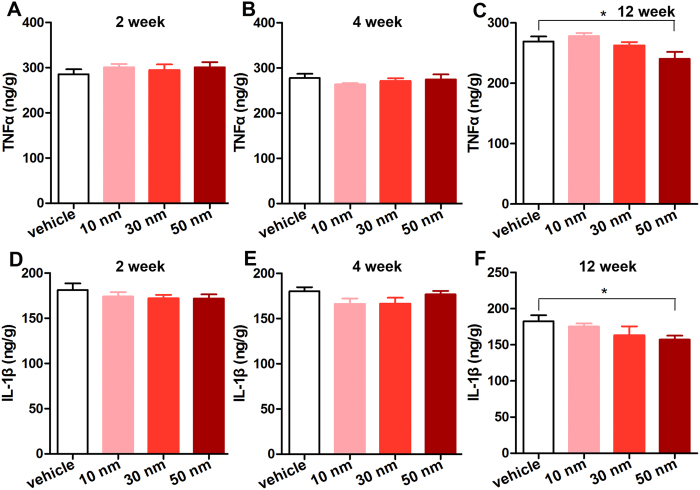
The concentration of TNF-α and IL-1β in the heart tissue. The concentration was assayed by ELISA. *P < 0.05. Data represent means ± SEM.

Furthermore, immunohistochemical (IHC) analysis was performed to detect inflammatory cells. It was reported that macrophages are central in eliminating GNPs^31^. Therefore, we detected macrophages in the heart sections of mice. Mac-3 positive (Mac-3^+^) cells were tested to determine the infiltration of macrophages. No Mac-3^+^ cells were detected in the heart sections of mice receiving 10, 30 and 50 nm GNPs at each time point (Fig. 7). Furthermore, immunohistochemical staining of CD45 positive (CD45^+^) cells in the heart sections was performed to determine the number of total inflammatory cells. The results showed that no CD45^+^ cells were observed either (Fig. 8; The positive control of inflammatory cell infiltration was exhibited in Fig. S1B,C). These data indicated that chronic GNPs exposure did not induce inflammatory cells infiltration in the heart. In addition, considering that inflammatory response may be manifested by increased cytokine, enzyme-linked immunosorbent assay (ELISA) was performed to analyze the level of TNF-α, which is a highly pleiotropic inflammatory mediator. There was no significant change in the level of TNF-α in the heart of mice receiving 10 and 30 nm GNPs (Fig. 9A,B). However, the mice receiving 50 nm GNPs showed a significant decrease in the TNF-α level compared with the vehicle group at the 12th week (238.30 ± 10.24 ng/g tissue vs. 269.50 ± 8.10 ng/g tissue, *P* < 0.05, Fig. 9C). Besides, the level of IL-1β, a proinflammatory mediator, was analyzed with ELISA. Interestingly, there was also a significant decrease in the IL-1β level in the heart of mice receiving 50 nm GNPs compared with the vehicle group at the 12th week (157.20 ± 5.44 ng/g tissue vs. 182.50 ± 8.33 ng/g tissue, *P* < 0.05, Fig. 9F). Taken together, these results showed that chronic GNPs exposure did not induce inflammatory cells infiltration in the mouse heart, whereas 50 nm GNPs led to a significant decrease in the TNF-α and IL-1β level in the heart of mice.

## Discussion

**Figure 10:**
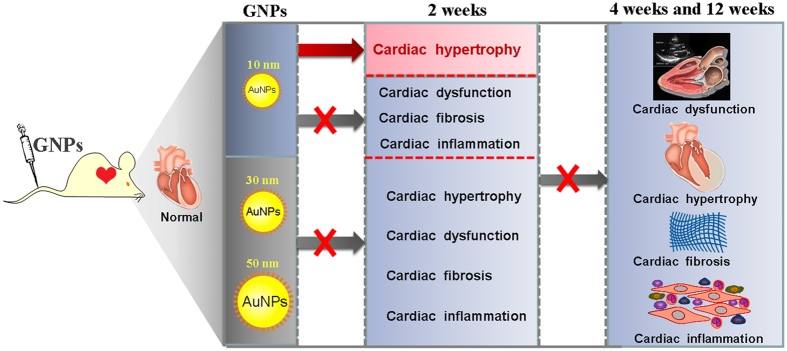
Working model of the effects of GNPs on the heart.

GNPs recently show promising application in drug delivery, molecular imaging, *in vivo* diagnosis and bio-sensing. And it’s well accepted that GNPs can be coated with PEG to add stability and protect against toxicity^32^. However, as engineered metallic particles, GNPs have gained much attention concerning their potential toxicity. In the present study, we found that 10 nm PEG-GNPs can cause reversible cardiac hypertrophy at the 2th week after repeated administration in mice. No overt cardiac systolic functional changes were detected after repeated PEG-coated gold nanoparticles administrations (10, 30, 50 nm) at different duration(2, 4 and 12 weeks). The three sizes of GNPs did not induce cardiac fibrosis or inflammatory cells infiltration. In contrast, 50 nm GNPs led to a significant decrease in the TNF-α, IL-1β and collagen I level in the heart of mice at the 12th week. These findings were summarized as Fig. 10.

It was reported that 12.5 nm GNPs did not induce mortality or any gross behavioral changes in mice at doses of 40, 200, 400 μg/kg · day daily for 8 days^33^. Our results also indicated that 10, 30 and 50 nm GNPs at the dose of 400 μg/kg · day daily for 14 days did not lead to mortality or overt behavioral changes on weeks 2, weeks 4 and weeks 12 after the administration of GNPs.

The heart is the center of the circulation system. Therefore, it’s pivotal to investigate the effects of GNPs on the heart. In the present study, we sought to provide a basic understanding of the chronic effects of GNPs on the heart. Previous studies indicated that the toxicity of GNPs may be related to particle sizes and accumulation time. Hence, three sizes of PEG-coated GNPs (10, 30 and 50 nm) were used and three time point (2, 4, and 12 week) were examined in our study. Our results indicated that the 10 nm GNPs showed highest accumulation in the heart at each time point, followed by 30 and 50 nm GNPs. Besides, the accumulation of 10 nm and 30 nm GNPs exhibited a decrease with increased duration. But the accumulation of 50 nm GNPs in the heart did not show overt change at the end of the study. The permeation of nanoparticles was reported highly dependent on the size of the nanoparticle in tumor^34^. Smaller GNPs rapidly diffused into the tumor matrix but did not retain for long, whereas larger GNPs stayed near the vasculature. In addition, it was reported that smaller GNPs exhibited wider biodistribution^21^,^35^. In consistent with these findings, we also found that GNPs accumulation depended the size and duration in the heart tissue. It’s well known that the function of the heart is to pump blood into the arteries. Thus, first of all, we examined the chronic effects of GNPs on the cardiac ejection fraction (EF) and fractional shortening (FS) *in vivo*. We found that the10, 30 and 50 nm PEG-coated GNPs did not affect the cardiac systolic function.

Furthermore, the cardiac structure was assessed. We found that the mice receiving 10 nm GNPs for 2 weeks increased statistically in HW/BW, LVMass and LVIDd. However, these changes were not found in the case of 30 and 50 nm GNPs or in mice receiving 10 nm GNPs for 4 and 12 weeks. The reason why 10 nm GNPs resulted in reversible cardiac hypertrophy could be: The given mass of the three sizes GNPs were identical. Because 10 nm GNPs were the least in the diameter, they were the most in the number. At the same mass, the molar concentration of the 10 nm GNPs is 27 and 125 times as high as the 30 nm and 50 nm GNPs respectively. It’s well known that the solutes in the blood vessel generate an important driving force– osmotic pressure, which is dependent on the molar concentration of solutes^36^. According to Starling’s law^37^, an increased osmotic pressure should favor blood volume expansion. Therefore, 10 nm GNPs may lead to an increase in blood volume by increasing crystalloid osmotic pressure. Then the increased blood volume resulted in cardiac hypertrophy.

In the present study, injury changes were not observed in the HE-stained sections of mouse heart. The immunohistochemical examination of heart sections also showed that GNPs did not induce inflammatory cell infiltration. These findings are consistent with the results of our previous study^20^. However, these data are inconsistent with the observations of Abdelhalim’s results, which showed GNPs induced heart muscle disarray with a few chronic inflammatory cells infiltrated and extravasation of red blood cells^18^. Two possible reasons may underlie the inconsistence. First, Wistar-Kyoto rats were employed in Abdelhalim’s study, while BALB/c mice were employed in our study. Different species may response differently to the similar treatment. Second, naked GNPs were used in Abdelhalim’s study, while PEG-coated GNPs were used in our study. PEG-coated GNPs has been shown to cause less immune response than the naked GNPs. These differences may cause different biological effects. In addition, Abdelhalim *et al.* only observed the HE-stained sections, while the immunohistochemical analysis was further performed in our study.

13 nm PEG-coated GNPs were reported to lead to acute inflammatory reaction in the liver^14^. However, in the present study, we found that chronic GNPs exposure did not induce inflammatory cells infiltration in the mouse heart. PEG-coated GNPs (50 nm) led to a significant decrease in the TNF-α and IL-1β level in the heart of mice at the 12th week. Interestingly, the mice receiving 50 nm GNPs also exhibited a significant decrease in the collagen I gene expression at the 12th week, while no overt cardiac fibrosis was detected in mice receiving 10, 30 and 50 nm GNPs using Sirius red staining at each time point. Considering all these results, chronic 50 nm GNPs exposure may serve as a strategy to inhibit inflammatory factors and collagen expression. After all, it has been a long history since gold is used to treat rheumatoid arthritis^5^,^38^,^39^. Besides, it’s well accepted that fibrosis is always in harmony with inflammatory response^40^.

## Methods

### Characterization of the GNPs

Three different sizes (10, 30 and 50 nm) of PEG-coated gold nanoparticles (GNPs) used in this experiment were from Nanocs Inc., New York (http://www.nanocs.com). The morphology, size and aggregation state of the GNPs were evaluated using transmission electron microscopy (TEM, JEM-200CX, Jeol Ltd., Japan) and Multiskan GO (Thermo Scientific, Ltdl, USA). The PEG-coated GNPs suspension was sonicated for 5 min before use.

### Animals

Our investigation was approved by the Biomedical Research Ethics Committee of Peking University (LA 2010-048) and strictly adhered to the American Physiological Society’s “Guiding Principles in the Care and Use of Vertebrate Animals in Research and Training”. 12-week-old male Balb/c mice were provided by the Animal Department of Peking University Health Science Center (Beijing, China). Mice were housed in groups of four and maintained on a 12 h dark/light cycle in a room with controlled temperature (25 ± 2 °C). Mice had free access to food and water.

The GNPs (400 μg/kg bodyweight) and vehicle control (PBS) were administered intravenously *via* tail vein for 14 consecutive days. Eight animals in each group were employed at each time point. The mice were sacrificed 2 weeks, 4 weeks and 12 weeks after the first injection. The hearts were excised for further studies.

### Echocardiographic analysis

Mice were anaesthetized using 1% isoflurane (Baxter Healthcare Corporation, New Providence, USA). Echocardiographic images were obtained using the Visualsonics high-resolution Vevo 770 system (VisualSonics, Incorporated, Toronto, Canada). Two-dimensional parasternal long-axis views and short-axis views were obtained at the level of the papillary muscle. The diastolic left ventricular posterior wall thickness (LVPW;d), systolic left ventricular posterior wall thickness (LVPW;s), diastolic left ventricular anterior wall thickness (LVPW;d), systolic left ventricular anterior wall thickness (LVPW;s), left ventricular end-diastolicinner-dimension (LVID;d) and left ventricular end- systolic inner-dimension (LVID;d) were measured. And the left ventricular mass (LVMass) were calculated from these parameters. All measurements were averaged from three consecutive cardiac cycles.

### Quantitative histological analysis

Following sacrifice, the hearts were harvested and perfused in retrograde with cold phosphate-buffered saline (PBS), fixed with 4% paraformaldehyde for 8 hours, dehydrated in 20% sucrose for 24 hours and then embedded in paraffin. Serial sections (5 μm thick) were stained with hematoxylin and eosin (H&E) for morphological analysis, and/or picrosirius red for the detection of fibrosis. For morphometrical analysis, photographs of left ventricular sections cut from the same location of each heart were observed under ×400 magnification (Leica Microsystems Imaging Solutions Ltd., Cambridge, UK). Interstitial fibrosis was visualized with picrosirius red staining.

### Immunohistochemistry analysis

Paraffin heart sections were deparaffinized in xylene and re-hydrated. Antigen retrieval was achieved by boiling the slides in citrate solution for 10 min and slides were then washed with PBS. After quenching endogenous tissue peroxidase activity with 3% H_2_O_2_ for 10 min, the slides were then washed in PBS and samples blocked in PBS containing 5% goat serum albumin at 37 °C for 1 hour. Primary antibodies to detect mouse CD45 (for hematopoietic stem cells and all cells of hematopoietic origin, except erythrocytes with rat anti-mouse CD45, 1:50 dilution, product no. 550539, BD Biosciences), mouse Mac-3 (for macrophages with rat anti-mouse Mac-3, 1:50 dilution, product no. 550292, BD Biosciences), were applied overnight at 4 °C in PBS. The samples were washed in PBS and then sequentially incubated with DAB Kit (ZSGB, Beijing, China) for 1 h in the next day. The peroxidase reaction was visualized using 3,3’-diaminobenzidine tetrahydrochloride (DAB) and slides were counterstained with Hematoxylin.

### Quantitative Real-Time PCR

Total RNA was isolated from heart tissue using Trizol Reagent (Invitrogen). The complementary DNA was synthesized according to the operating instruction (Promega, Madison, WI, USA). Relative quantitation by real-time PCR was performed using SYBR Green to detect PCR products in real time with ABI PRISM 7700 Sequence Detection System (Applied Biosystems). The real-time PCR primer sequences for GAPDH (glyceraldehyde 3-phosphate dehydrogenase) were forward: 5′-ATGTTCCAGTATGACTCCACTCACG-3′ and reverse: 5′-GAAGACACCAGTAGACTCCACGACA-3′. The primer sequences for collagen I were forward 5′-GTAACTTCGTGCCTAGCAACA-3′ and reverse 5′- CCTTTGTCAGAATACTGAGCAGC-3′. PCR was performed under the following conditions: 95 °C for 2 min and 40 amplification cycles (92 °C for 15 s, 55 °C for 15 s and 72 °C for 30 s). The CT (threshold cycle) values obtained for genes of interest were normalized to concurrent measurement of GAPDH mRNA levels, and fold changes were compared with controls.

### Enzyme-linked immunosorbent assay (ELISA)

Approximately 40 mg of cardiac tissue from the same part of the heart was transferred to a tube, homogenized in 800 μl lysis buffer, and then centrifuged at 12,000 rpm for 15 min at 4 °C temperature, after which the upper layer was collected for further analysis. The protein concentration was estimated by BCA protein assay kit (Pierce, Rockford, IL, USA). The concentrations of soluble inflammatory cytokines were measured by commercially available ELISA kits (4A Biotech, Beijing, China) according to the manufacturer’s instructions.

### ICP-MS

The tissue concentrations of GNPs were assessed by quantitative inductively coupled plasma mass spectrometry (ICP-MS). Approximately 30 mg of cardiac tissue were digested in aqua fortis (nitric acid: hydrochloric acid 3:1). After adjusting the solution volume to 2 ml using 2% nitric acid and 1% hydrochloride acid (1:1), Au content assays were performed using an ELAN DRC e ICP-MS instrument (Perkin Elmer, Massachusetts, USA).

### Statistical analysis

Data are summarized as means ± SEM. Differences in data among groups were compared with one-way ANOVA followed by Dunnett’s test using Prism 5 (GraphPad Software Incorporated, La Jolla, CA, USA). *P* < 0.05 was considered statistically significant.

## Additional Information

**How to cite this article**: Yang, C. *et al.* Reversible cardiac hypertrophy induced by PEG-coated gold nanoparticles in mice. *Sci. Rep.* **6**, 20203; doi: 10.1038/srep20203 (2016).

## Supplementary Material

Supplementary Information
