# Supplementary material for: Reversible cardiac hypertrophy induced by PEG-coated gold nanoparticles in mice
Source: Sci Rep. 2016 Feb 1;6:20203. doi: 10.1038/srep20203 (PMC4735330; doi:10.1038/srep20203)
Supplement: Supplementary Information [file srep20203-s1.pdf]

Reversible cardiac hypertrophy induced by PEG-coated gold nanoparticles in mice

Chengzhi Yang, Aiju Tian, Zijian Li*

Institute of Vascular Medicine, Peking University Third Hospital, Key Laboratory of Cardiovascular Molecular Biology and Regulatory Peptides, Ministry of Health, Key Laboratory of Molecular Cardiovascular Sciences, Ministry of Education and Beijing Key Laboratory of Cardiovasicular Receptors Research Beijing 100191, China

* Corresponding author: Zijian Li, Tel.: +86 10 82265519, E-Mail: lizijian@bjmu.edu.cn.

Supplementary figure:

Figure S1.

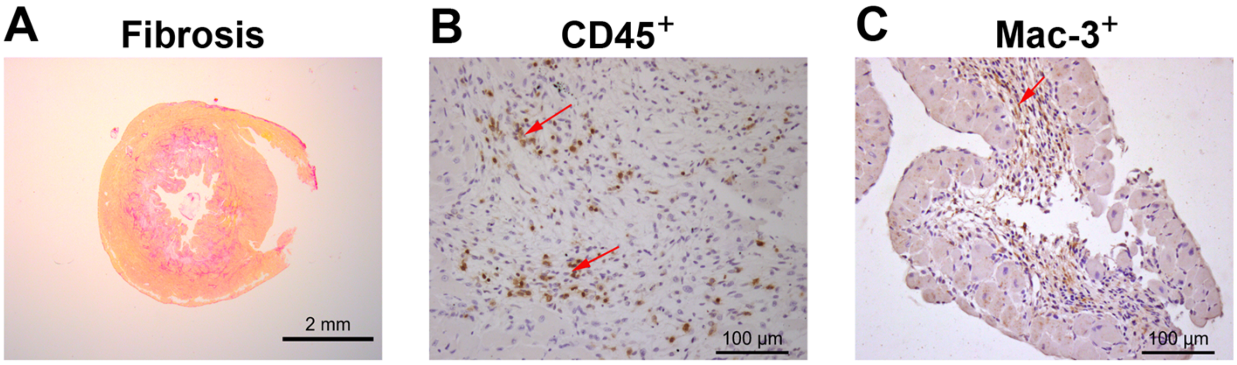

Supplementary figure legend:

Figure S1. The positive control in the experiment. The heart sections of positive control were from Balb/c mice receiving subcutaneous injection of isoproterenol (5 mg/Kg•day, dissolved in saline, Sigma-Aldrich, St. Louis, USA) for 7 consecutive days. (A) Representative micrographs of picrosirius red-stained sections of the heart. Red parts represent collagen. The scalebar is 2mm. (B) Representative image showing CD45+cells in the immunohistochemical (IHC) analysis. Arrows indicate brown areas with CD45+cells. (C) Representative micrographs showing Mac-3+cells. Arrows indicate brown areas with Mac-3+cells. The scalebars are 100μm.
